# Sequential and co-occurring DNA damage response genetic mutations impact survival in stage III colorectal cancer patients receiving adjuvant oxaliplatin-based chemotherapy

**DOI:** 10.1186/s12885-021-07926-1

**Published:** 2021-03-02

**Authors:** Peng-Chan Lin, Yu-Min Yeh, Ren-Hao Chan, Bo-Wen Lin, Po-Chuan Chen, Chien-Chang Pan, Meng-Ru Shen

**Affiliations:** 1 Department of Oncology, National Cheng Kung University Hospital, College of Medicine, National Cheng Kung University, Tainan, Taiwan; 2 Department of Genomic Medicine, National Cheng Kung University Hospital, College of Medicine, National Cheng Kung University, Tainan, Taiwan; 3 Department of Computer Science and Information Engineering, College of Electrical Engineering and Computer Science, National Cheng Kung University, Tainan, Taiwan; 4 Department of Surgery, National Cheng Kung University Hospital, College of Medicine, National Cheng Kung University, Tainan, Taiwan; 5 Department of Pharmacology, National Cheng Kung University Hospital, College of Medicine, National Cheng Kung University, Tainan, Taiwan; 6 Graduate Institute of Clinical Medicine, National Cheng Kung University Hospital, College of Medicine, National Cheng Kung University, Tainan, Taiwan; 7 Department of Obstetrics and Gynecology, National Cheng Kung University Hospital, College of Medicine, National Cheng Kung University, No.138, Sheng-Li Road, Tainan, 704 Taiwan

**Keywords:** Sequential somatic mutations, DNA damage response gene, Colorectal cancer, Tumor heterogeneity, Tumor evolution

## Abstract

**Background:**

Certain sequences of genomic mutations can lead to cancer formation and affect treatment outcomes and drug resistance. We constructed a cancer evolutionary tree using bulk-targeted deep sequencing to explore the impact of sequential and co-occurring somatic mutations on patients with stage III colorectal cancer (CRC).

**Methods:**

A total of 108 stage III CRC patients from National Cheng Kung University Hospital (NCKUH) were recruited for this study between Jan. 2014 and Jan. 2019. Clinical information and tumor-targeted deep sequencing data were collected. Phylogenetic trees were reconstructed for evolutionary trajectories. We used a machine learning model for survival analysis.

**Results:**

Six sequential somatic mutations stratified patients into seven subgroups based on survival. Patients carrying sequential germline followed by DNA damage response-related *ATM* or *BRCA2* somatic mutations or non-*TP53, APC* somatic mutations had a better outcome than those without such mutations. The 4-year recurrence-free survival (RFS) probability was 88% in the low-risk group (G1) and 46% in the high-risk group (G2) (log-rank *p*-value 2e-05). The predictive efficacy by the area under the curve (AUC) was 0.73, 0.7, 0.797, and 0.88 at 2, 4, 6, and 8 years, respectively. The mutation status of mismatch repair (MMR) genes was not associated with RFS. Different genomic features were found between the groups. The orders of *APC*, *KRAS* and *APC*, *BRCA2* sequential somatic mutations were associated with clinical outcomes. The occurrence of somatic mutations in *BRCA2*, such as *TP53* somatic mutations, affected recurrence-free survival.

**Conclusions:**

According to the evolution model, DNA damage response (DDR)-related *ATM* or *BRCA2* somatic mutations are promising biomarkers for assessing the response of stage III CRC patients to oxaliplatin-based chemotherapy. The sequential order and co-occurring DDR somatic mutations are associated with recurrence-free survival.

**Supplementary Information:**

The online version contains supplementary material available at 10.1186/s12885-021-07926-1.

## Background

Patients with cancers that are associated with high intratumor heterogeneity might experience poor clinical outcomes [1]. As a result of this heterogeneity, the bulk tumor might include a diverse group of cells harboring distinct molecular signatures. Despite the success of targeted therapies for cancer, the development of resistance limits the ability to translate this method into a curative treatment. One possible resistance mechanism has traditionally been thought of as the intrinsic co-occurrence of acquired genetic mutations [2, 3].

Certain sequences of genomic mutations can lead to cancer development and affect treatment outcomes and drug resistance. Phylogenetic evolutionary trees with different genetic mutation orders may reveal information on cancer biology that can be used to plan therapeutic strategies [4, 5]. In a mouse model, we found that the temporal sequence of two mutations might impact tumorigenesis due to different sequential orders of *RAS* and *TP53* somatic mutations and practical cooperation with other genetic mutations in cell line models [6, 7]. Clinically, *EGFR*-mutant/*TP53*-mutant lung adenocarcinoma should be regarded as a unique subgroup with a poor prognosis [8]. Studies have implied that the genetic mutation order or co-occurring genetic mutations are important in cancer biology.

In Pan-Cancer Atlas datasets, there are 141/528 cases of putative driver mutations in 29 genes associated with the DNA damage response (DDR) and DNA repair (including the mismatch repair and homologous recombination pathways) [9, 10]. For example, poly (ADP)-ribose polymerase inhibitors have shown promising results in preclinical studies on colorectal cancer (CRC) patients carrying *BRCA1*/*BRCA2* mutations. Other DNA repair-targeting therapies, such as *ATR* and *CHK1* inhibitors, which are most effective against cancers carrying *ATM* mutations, can be applied along with current genotoxic chemotherapy in CRC [11]. Targeting alternative DDR mechanisms may also improve the clinical outcomes of CRC patients.

In this study, we constructed a cancer evolutionary tree using bulk-targeted deep sequencing. We also explored the impact of DDR genes on stage III CRC patients and evaluated the effect of sequential and co-occurring somatic mutations.

## Methods

### Enrollment of patients

A total of 108 CRC patients from National Cheng Kung University Hospital (NCKUH) were recruited for this study between Jan. 2014 and Jan. 2019. All CRC cases were at pathological stage III and were treated with standard surgical resection followed by adjuvant chemotherapy with the mFOLFOX6 regimen (5-fluorouracil, leucovorin, and oxaliplatin). Clinical information was obtained from medical records. Tumor tissues and blood samples were collected at the time of enrollment. This study was approved by the Institutional Review Board of NCKUH (A-ER-103-395 and A-ER-104-153) and conducted according to the principles of the Declaration of Helsinki. All participants provided written informed consent.

### Targeted tumor sequencing with a cancer panel

A total of 108 primary tumor samples were subjected to histological assessment, followed by nucleic acid extraction from formalin-fixed paraffin-embedded blocks at NCKUH. Pathologists reviewed the specimens and determined the percentage of viable tumor nuclei and the adequacy of the sample for mutational profile detection. Targeted deep sequencing of tumor samples was performed with Oncomine Comprehensive Assays (OCAs) (Thermo Fisher Scientific). All samples were analyzed using Torrent Suite Software 5.0.4. All reads were aligned to the hg19 reference genome, and variant calling was performed using the Torrent Variant Caller plugin (version 5.0.4.0).

### Phylogenetic tree construction and evolutionary trajectory analysis

All somatic events (single-nucleotide variants (SNVs)/indels) were used to build a phylogenetic tree for each patient. Somatic SNVs with a minor allele frequency (MAF) less than 0.1% were filtered with ExAC EAS and in-house Taiwan Biobank WGS data. Driver genes were defined as nonsynonymous or indel variants; nondriver genes were defined as other intronic or synonymous variants. A clonal inference analysis of each patient was performed using the R package sciClone [12]. Phylogenetic trees and repeated evolutionary trajectories were produced with the R package Revolver [13]. Only driver genes were analyzed, and those that only occurred once were ignored.

### Statistical analysis

The chi-square test or Fisher’s exact test was used to determine the association between CRC patients’ clinical characteristics and survival. Kaplan–Meier curves and Cox proportional hazards models were employed to evaluate recurrence-free survival (RFS), which was defined as the time between surgery and cancer recurrence. A *p*-value adjusted by a false discovery rate (FDR) or a Bonferroni (BF) critical value of < 0.05 was considered statistically significant. All statistical analyses were conducted in the R environment.

### Machine learning model and analysis

#### CRC survival decision tree

The CRC survival decision tree was built with the package Recursive Partitioning and Regression Trees (rpart) [14] and visualized with partykit [15]. We set the parameter minsplit to 30, with each node containing at least ten patients. The 108 CRC patients were divided into seven subgroups.

#### Feature selection

We used the least absolute shrinkage and selection operator (LASSO) regression algorithm for feature selection. To obtain more specific features, we implemented the algorithm 100 times and used all 22 features.

## Results

### Clinical features and genomic phylogenetic mutations

**Fig. 1:**
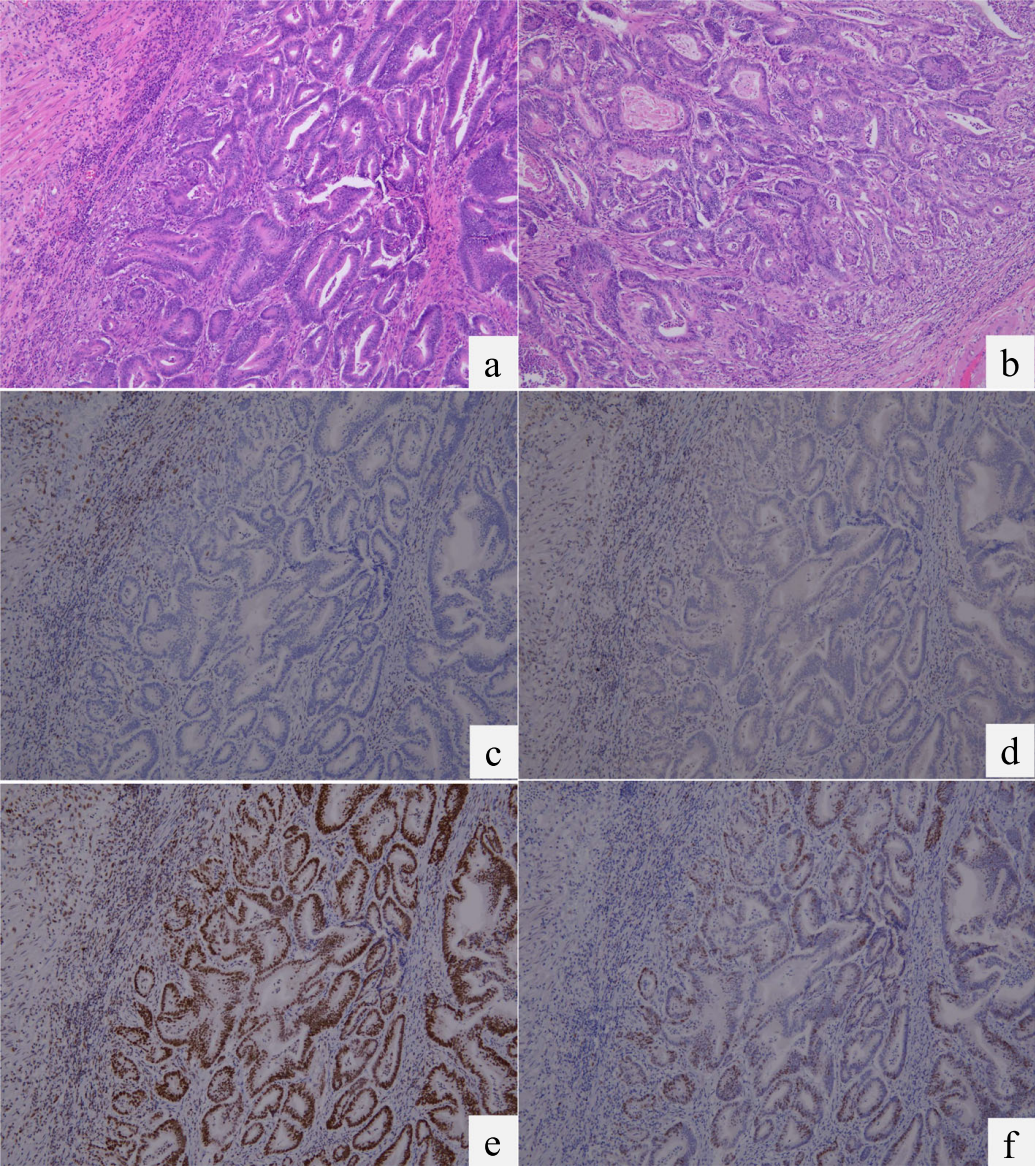
An example of dMMR colorectal cancer stained with hematoxylin-eosin (**a, b**). The cancer cells revealed loss of nuclear staining of *MLH1* (**c**) and *PMS2* (**d**), and preserved nuclear staining of *MSH2* (**e**) and *MSH6* (**f**) (100x)

We enrolled 108 stage III colorectal cancer (CRC) patients in this study. For all subjects, we collected their primary tumor tissues for targeted deep sequencing. The baseline characteristics of the patients are provided in Supplementary Table S1. There was an equal sex distribution among the patients. The median patient age was 57.5 years (interquartile range (IQR), 49.75–65.0 years). Sex ratios were equal (50%, *N* = 54). The LENT primary tumor site was the left colon (75.9%, *N* = 82). Most of the cases were at a highly invasive tumor stage (T3/T4 stage) (87%, *N* = 94) but a low lymph node stage (N0/N1 stage) (71.3%, *N* = 77). Microsatellite instability (MSI) high, or DNA mismatch repair (MMR) protein loss by immunohistochemistry staining of tumors (mismatch repair deficiency (dMMR)) (5.6%, *N* = 6) was detected in only a few patients. Immunohistochemical (IHC) expression of MMR proteins was interpreted by the same pathologist in our institute. An example of MMR protein expression in colonic adenocarcinoma tissues is shown in Fig. 1, hematoxylin and eosin staining (H&E × 100) with moderately differentiated carcinoma in Fig. 1a and b, loss of *MLH1* and *PMS2* expression in Fig. 1c and d, and positive IHC expression of *MSH2* and *MSH6* in Fig. 1e and f. Positive MSI markers were reexamined to confirm the results.

The mutational status of mismatch repair genes, including *MLH1* and *MSH2,* in all patients was analyzed. Twenty-two of the 108 CRC patients showed MMR genetic variants. The association of group 1 (G1) and group 2 (G2) subjects with MMR genetic variants was tested by Fisher’s exact test (*p*-value = 0.99). (Supplementary Table S1). A heatmap of MMR and homologous recombination genetic variants is shown in Supplementary Fig. S1. The association of *MLH1* and *BRCA2* genetic variants was mutually exclusive, whereas others coexisted (Supplementary Table S2).

**Fig. 2:**
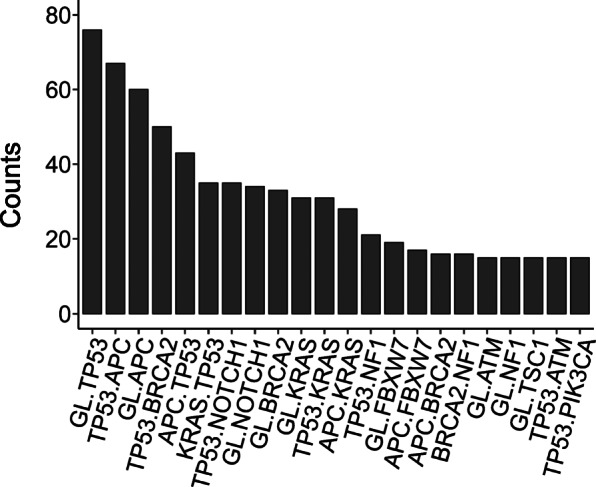
The spectrum and frequency of sequential somatic mutations in 108 CRC patients. The most common sequential somatic mutation was germline mutation. *TP53* occurred 76 times in our cohort. *TP53*, *APC* occurred 67 times. Germline, *APC* occurred 60 times. Only sequential somatic mutations that occurred at least 15 times are shown in the plot

To understand each patient’s sequential somatic mutations, we constructed evolutionary trees and extracted sequential somatic mutations with the Revolver tool [13]. Overall, 302 sequential somatic mutations were detected. Each CRC patient showed an average of 18 sequential somatic mutations, and the distribution of sequential somatic mutations ranged from 2 to 107. The spectrum and frequency of sequential somatic mutations are depicted in Fig. 2. Germline followed by *TP53* (Germline, *TP53*) was the most common sequential somatic mutation (70.4%). Other sequential somatic mutations were *TP53* followed by *APC* (*TP53*, *APC*) (62%) and germline followed by *APC* (Germline, *APC*) (55.6%). A bar plot of the spectrum of sequential somatic mutations occurring at least 15 times was constructed.

### Survival stratification and predictive efficacy according to mutation of DNA damage genes

**Fig. 3:**
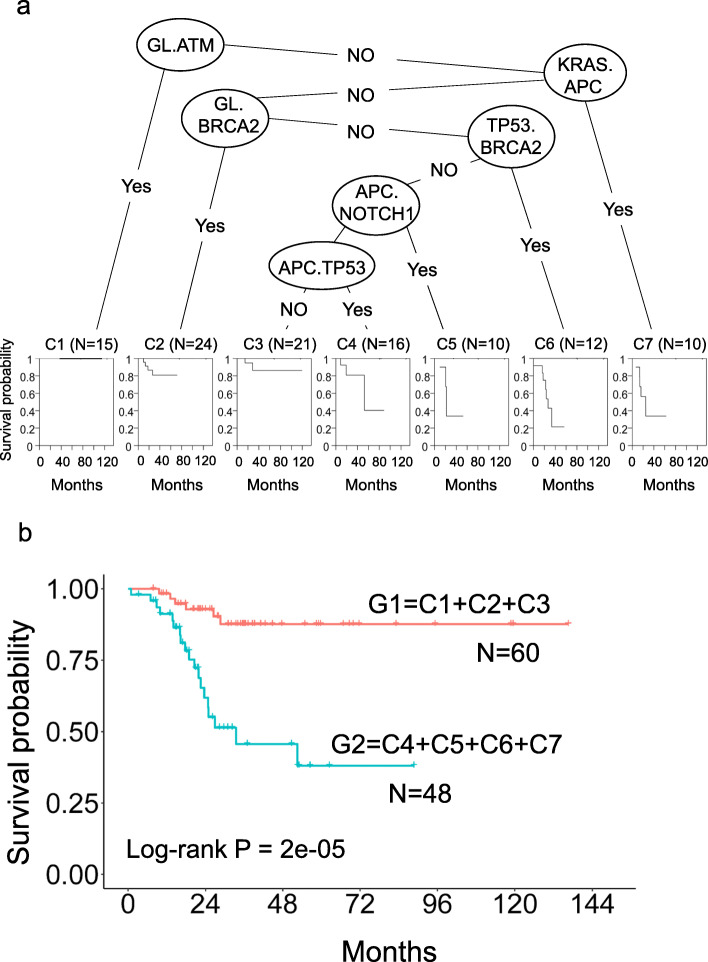
CRC survival decision tree analysis. **a** A decision tree was generated to optimize risk stratification in our cohort. The internal nodes of the tree represent sequential somatic mutations. The leaf nodes represent recurrence-free survival for each subgroup, and each node is representative of at least ten patients. **b** Kaplan–Meier plot of the decision subgroups. Patients in the G1 subgroup had a higher probability of 4-year recurrence-free survival (probability = 0.88, 95% CI = 0.79–0.98) than patients in the G2 subgroup (probability = 0.46, 95% CI = 0.30–0.69). The hazard ratio was 5.80 (95% CI = 2.30–14.61), and the log-rank *p*-value was 2e-05 (median recurrence-free survival, NA months [95% CI, NA-NA] vs 33.47 months [95% CI, 23.63-NA] (G1 = C1 + C2 + C3, G2 = C4 + C5 + C6 + C7)

Recursive partitioning analysis (RPA) was implemented to construct a survival decision tree to further improve CRC risk stratification according to sequential somatic mutations. As a result, the survival decision tree was generated with six sequential somatic mutations that separated the patients into seven subgroups (C1–7). The patients in subgroups C1, C2, and C3 had a better survival rate than the patients in subgroups C4, C5, C6, and C7 (Fig. 3a). Patients carrying germline followed by *ATM, BRCA2* or non-*TP53 APC* somatic mutations had a better outcome than those without such mutations (Fig. 3b). The *ATM* and *BRCA2* somatic mutation sites are shown in Supplementary Table S3. DNA damage response (DDR) somatic mutations are promising biomarkers to assess the response of stage III CRC patients to oxaliplatin-based chemotherapy.

We also classified patients in C1–3 as the low-risk group (G1) and patients in C4–7 as the high-risk group (G2). The clinical characteristics of the low-risk (G1) and the high-risk (G2) groups were not significantly different (Supplementary Table S1). The clinical characteristics of the DDR mutation group (C1–2), the no mutation group (C3), and the high-risk group (C4–7) also did not differ significantly (Supplementary Table S4). Kaplan–Meier curve analysis between the low-risk group (G1) and the high-risk group (G2) showed a significant difference (log-rank *p*-value 2e-05), and the 4-year recurrence-free survival probability was 88% in the G1 group and 46% in the G2 group (Fig. 3b). The median RFS was not reached in the low-risk group, and it was 33.5 months in the high-risk group. There was no statistically significant difference in survival between the DDR mutation subgroups and the no mutation subgroup (Supplementary Fig. S2). To assess predictive efficacy, time-dependent receiver operating characteristic (ROC) curves for 2, 5, 6, and 8 years before recurrence for the 2 groups were examined, and the area under the curves (AUCs) in the training set were 0.73, 0.7, 0.797, and 0.88, respectively. These results are shown in Supplementary Fig. S3.

Based on mutations in DDR genes (Fig. 3), we found co-occurrence of *BRCA2* and *TP53* somatic mutations to be associated with a poor prognosis. The median survival duration was 26.9 months. These results suggest the importance of the occurrence of somatic mutations.

### Multivariate analysis of the clinical and prognostic significance of genomic groups

**Fig. 4:**
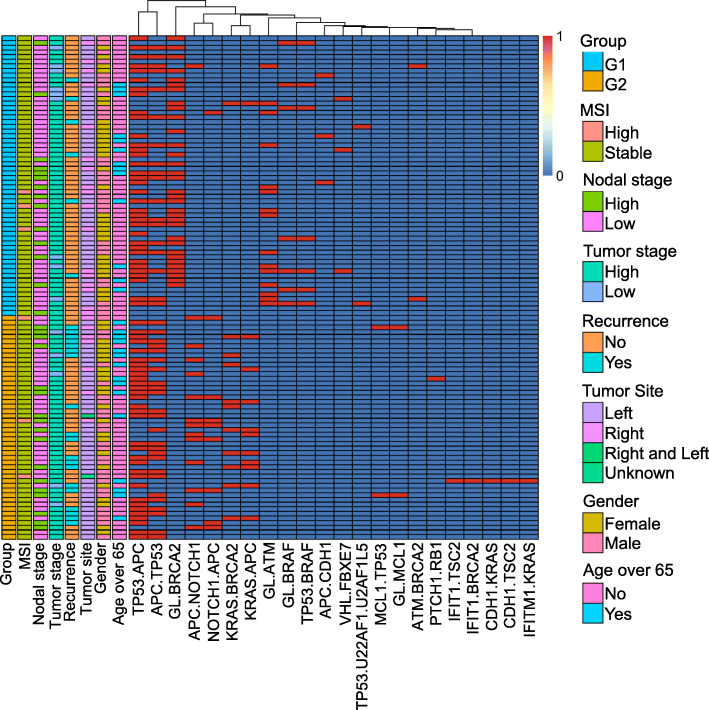
Heatmap of CRC sequential somatic mutations and clinical characteristics. We divided the patients into two subgroups (G1 and G2). Patients with a better prognosis in the previous survival decision subgroup (C1, C2, and C3) formed subgroup G1; the patients with a worse prognosis (C4, C5, C6, and C7) formed subgroup G2. We show only the 22 sequential somatic mutations that distinguished the two subgroups based on LASSO regression

The LASSO regression model was implemented to distinguish sequential somatic mutations between the low-risk group (G1) and the high-risk group (G2). We selected 22 sequential somatic mutations with the bootstrap method. A heatmap of the hierarchical clustering of the sequential somatic mutations and their clinical characteristics is provided in Fig. 4. We also used principal component analysis (PCA) to evaluate the tendency of the critical features. As a result, germline mutations followed by *BRCA2*, *ATM* and *BRAF* somatic mutations were unique to the low-risk group (G1); *APC*, *TP53* and *APC*, *NOTCH1* somatic mutations were unique to the high-risk group (G2) (Fig. 4 and Supplementary Fig. S4). A heatmap of sequential somatic mutations occurring at least ten times is shown in Supplementary Fig. S5.

Next, we performed univariate and multivariate analyses of recurrence-free survival. However, univariate and multivariate Cox regression models revealed no association between patient age, sex, primary tumor location site, tumor invasion, lymph node status, mismatch repair status, or MMR genetic variants and recurrence-free survival (RFS) (Table 1 and Supplementary Fig. 1). Nevertheless, a significant difference in RFS between the low-risk group (G1) and the high-risk group (G2) was observed in both models (adjusted hazard ratio (HR) = 7.46, 95% confidence interval (CI) = 2.48–18.56, *p*-value = 0.000192) (Table 1).

**Table 1:** Univariate and multivariate analysis

Characteristic		Univariate analysis		Multivariable analysis	
		HR(95%CI)	*P* value	HR(95%CI)	*P* value
Age	< 65 vs > =65	1.48 (0.64–3.45)	0.361	1.3 (0.55–3.07)	0.56
Gender	Female vs Male	0.78 (0.35–1.71)	0.534	0.72 (0.31–1.66)	0.44
Site	Left vs Right	0.85 (0.29–2.49)	0.769	1.81 (0.53–6.16)	0.34
T	T1/T2 vs T3/T4	1.08 (0.37–3.16)	0.887	1.11 (0.36–3.45)	0.853
N	N0/N1 vs N2	0.68 (0.30–1.53)	0.347	0.82 (0.35–1.92)	0.649
Mismatch Repair Status	Proficient vs Deficient	–	0.997	–	–
MMR genetic variants	Mutation vs Wild	1.03 (0.39–2.7)	0.954	0.94 (0.34–2.61)	0.914
Risk group	G1 vs G2	5.80 (2.30–14.61)	0.000187	7.46 (2.48–18.56)	0.000192

Abbreviations: *T* tumor invasion, *N* lymph node, *MSI* microsatellite instability, *G1* low-risk group, *G2* high-risk group, *HR* hazard ratio, *MMR* mismatch repair

### The order of sequential somatic mutations is associated with clinical outcomes

**Fig. 5:**
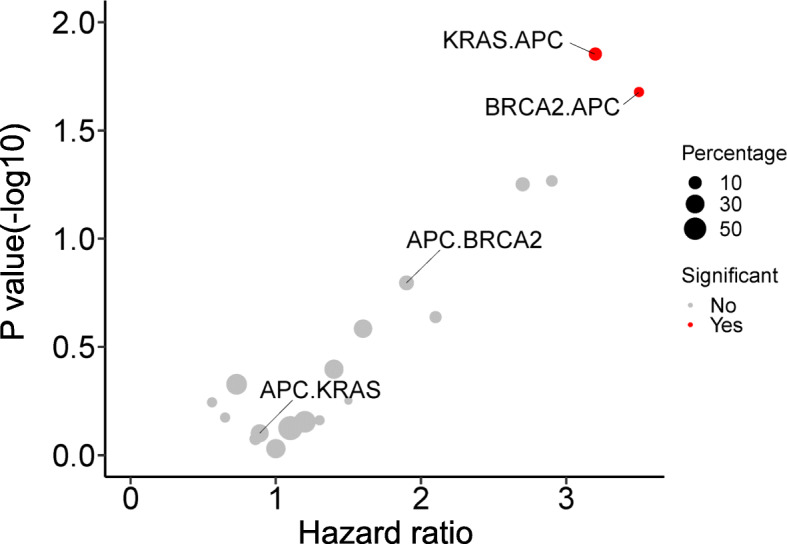
Scatter plot of sequential somatic mutations associated with clinical outcomes. The sequential genetic order of *KRAS*, *APC* with *APC*, *KRAS* and *BRCA2*, *APC* with *APC*, *BRCA2* has a different impact on recurrence-free survival. *KRAS*, *APC* and *BRCA2*, *APC* were associated with RFS; *APC*, *KRAS* and *APC*, *BRCA2* were not. The *p-*value was calculated using the hazard ratio. Red dots represent the statistical significance of the hazard ratio, and the size of the circle represents the percentage of patients with sequential somatic mutations

To determine whether the order of somatic mutations impacts recurrence-free survival, we examined sequential somatic mutations in two genetic orders: reverse and inverse. As a result, we found that the frequency of sequential *KRAS* followed by *APC* somatic mutations (*KRAS*, *APC*) was 10.2% and that of sequential *BRCA2*, *APC* somatic mutations was 5.6%; both were associated with a poor prognosis (hazard ratio (HR) = 3.2, 95% confidence interval (CI) = 1.3–7.99, *p*-value = 0.01, and HR =3.5, 95% CI = 1.2–10.31, *p-*value = 0.02). In contrast, there was no statistically significant difference in survival in the inverse order of sequential *APC*, *KRAS* somatic mutations (25.9%) and *APC*, *BRCA2* mutations (14.8%) (HR = 0.89, 95% CI = 0.37–2.13, *p-*value = 0.8 and HR = 1.9, 95% CI = 0.78–4.47, *p-*value = 0.2). The results are illustrated as a scatterplot in Fig. 5.

**Fig. 6:**
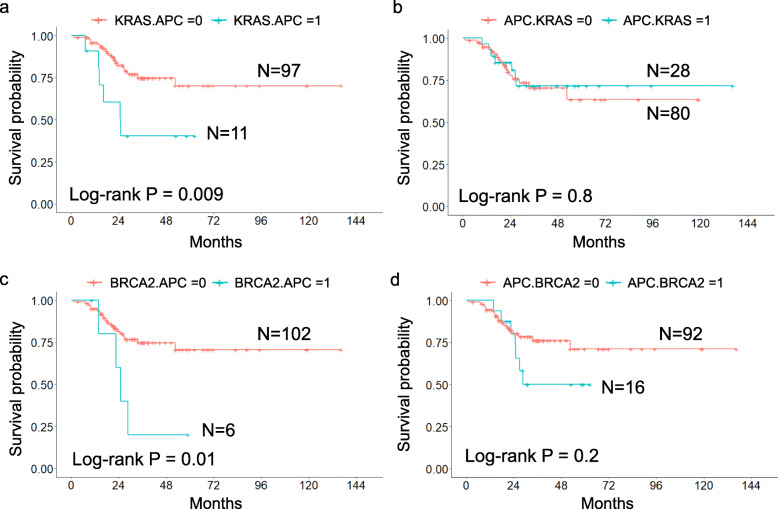
Kaplan–Meier plot of sequential genetic mutations with different orders. **a** Kaplan–Meier analysis of patients with sequential *KRAS*, *APC* somatic mutations revealed a significantly poor survival outcome, with log-rank *p-*values of 0.009. **b** There were no significant differences in patients with sequential genetic *APC*, *KRAS* somatic mutations (*p-*values of 0.8). **c** *BRCA2*, *APC* somatic mutations also led to a significantly poor survival outcome, with log-rank *p-*values of 0.01. **d** There were no significant differences in patients with *APC*, *BRCA2* somatic mutations (*p-*values of 0.2). CRC patients with the sequential *KRAS*, *APC* or *BRCA2*, *APC* somatic mutations had a worse prognosis than patients without sequential somatic mutations. Sequential *APC*, *KRAS* and *APC*, *BRCA2* somatic mutations were not associated with recurrence-free survival. The 4-year probability of RFS according to *KRAS*, *APC* somatic mutations (probability = 0.40, 95% CI = 0.19–0.86) were less than *APC, KRAS* somatic mutations (probability = 0.72, 95% CI = 0.55–0.92). The 4-year probability of RFS according to *BRCA2*, *APC* somatic mutations (probability = 0.20, 95% CI = 0.03–1) were less than *APC, BRCA2* somatic mutations (probability = 0.5, 95% CI = 0.29–0.85)

Kaplan–Meier analysis of patients (Fig. 6 a and c) with sequential *KRAS*, *APC* or *BRCA2*, *APC* somatic mutations also revealed a significantly poor survival outcome, with log-rank *p-*values of 0.009 and 0.01, respectively. When sequential somatic mutations were reversed (*APC*, *KRAS* somatic mutation and *APC*, *BRCA2* somatic mutation), there were no significant differences in the Kaplan–Meier curves, with *p-*values of 0.8 and 0.2, respectively (Fig. 6 b and d). The 4-year recurrence-free survival probabilities for patients with *KRAS*, *APC* and *BRCA2*, *APC* somatic mutations were 40 and 20%, respectively, which were significantly lower than those for patients with *APC*, *KRAS* and *APC*, *BRCA2* somatic mutations (72 and 50%, respectively) (Fig. 6). The median RFS duration in patients with sequential *KRAS*, *APC* somatic mutations was not reached, whereas it was reached for patients with *APC*, *KRAS* somatic mutations, at 24.9 months. Median RFS durations for patients with sequential *BRCA2*, *APC* and *APC*, *BRCA2* somatic mutations were 24.9 and 28.6 months, respectively.

## Discussion

With advances in NGS technology, tumor-targeted sequencing is becoming an affordable tool for patients with cancer. Indeed, these advances enhanced our ability to identify the impact of tumor heterogeneity and evolution when developing drug response genetic markers in our previous study [16]. To address these issues, we performed a cancer genomics evolutionary trajectory analysis. Our results are highlighted as follows: (i) mutations in DNA damage genes, such as *ATM* or *BRCA2* somatic mutations, can impact the clinical outcomes of stage III CRC patients; (ii) we divided patients into low-risk (G1) and high-risk (G2) groups to predict survival based on sequential somatic mutations; and (iii) the order and occurrence of sequential somatic mutations are associated with clinical outcomes.

Mutations in DNA damage repair (DDR) genes, such as those involved in mismatch repair (MMR), nucleotide excision repair (NER), homologous recombination (HR), Fanconi anemia (FA), and checkpoints, play an important role in DNA damage repair, and defects in these genes may predict the response to platinum-based chemotherapy [11, 17]. Currently, colorectal cancer patients receive an adjuvant chemotherapy mFOLFOX6 regimen with 5-fluorouracil, leucovorin, and oxaliplatin. 5-Fluorouracil (5-FU) is an antimetabolite drug that inhibits thymidylate synthase and is thought to inhibit DNA replication [11, 18]. 5-FU is also incorporated into DNA, causing DNA mismatches to be recognized and repaired by the mismatch repair (MMR) pathway [19]. Oxaliplatin is a platinum-based compound that induces cell death through several mechanisms [20]. Oxaliplatin causes DNA interstrand cross-link damage that is repaired by nucleotide excision repair proteins. This type of damage is repaired during G1 and by the Fanconi anemia and HR proteins during S phase [18, 21].

Mismatch repair (MMR) status can be used for clinical decision-making for patients with stage II colon cancer receiving 5-FU chemotherapy regimen. However, colon cancer patients in stage III with MMR-proficient tumors have clinical outcomes similar to those of MMR-deficient patients [22]. As MMR status was not associated with the prognosis of stage III colon cancer patients, we used other DNA damage response (DDR) somatic mutations in our study. Precision treatment strategies also target tumor-specific DDR somatic mutations [23]. In a previous study, *ATM* defects were detected in 15% of CRC patients and were associated with improved overall survival [24]. *BRCA* mutations can help personalize patient treatment by potentially adding platinum/DNA-damaging agents or PARP inhibitors [25]. Our study is notable because a significant survival advantage was observed in stage III CRC patients with sequential DDR gene mutations (39/108 (36.1%) patients in our cohort). The germline mutation followed by *ATM* and *BRCA2* somatic mutations was found in 15/108 (13.8%) and 24/108 (22.2%) patients, respectively. Although the median RFS duration for the entire cohort was not reached, the occurrence of mutations in *BRCA2*, such as *TP53* somatic mutation, resulted in different survival rates, as did different sites of the primary tumor (patients with a tumor on the left side of the colon and those with DDR gene mutations; *p-*value = 0.017). Our results show that DDR somatic mutations and co-occurring somatic mutations play an important role in clinical outcomes. We used cBioPortal, a web platform for gene-based data exploration, to demonstrate proof of concept. Based on CRC genomic data, we found that *ATM* mutations were associated with better survival. The presence of *BRCA1* and *TP53* somatic mutations resulted in a poorer prognosis than *BRCA1* somatic mutations only in CRC patients. The results are shown in Supplementary Fig. S6.

Oxaliplatin-containing combination chemotherapy, including the FOLFOX regimen, is a well-established standard of care for stage III CRC patients receiving adjuvant therapy. Nonetheless, side effects such as neuropathy can negatively influence the quality of life. Interestingly, we built a survival prediction model for recurrence according to six sequential somatic mutations. Multivariate analysis revealed that patients in the high-risk group (G2) had a higher recurrence rate. These results suggest that we should consider another therapeutic strategy for patients at a high risk of recurrence.

Cancers result from the accumulation of somatic mutations, and their properties are thought to reflect the sum of these mutations. The order in which somatic mutations are acquired determines how individual cancers behave [4, 5], and the order in which *JAK2* and *TET2* are mutated influences clinical features and response to targeted therapy in patients with myeloproliferative neoplasms [26]. Compared with patients in whom *TET2* mutation was acquired first, patients in whom the *JAK2* mutation was acquired first had an increased risk of thrombosis and a poor prognosis. Overall, the tumor phenotype is directly influenced by different sequential orders of *RAS* and *TP53* somatic mutations [6]. Patients harboring *EGFR* and comutation of tumor suppressor genes have poor recurrence-free survival [7, 8]. These studies indicate that sequential or co-occurring somatic mutations are important in cancer biology. In our study, sequential *KRAS* and *APC* somatic mutations as well as sequential *BRCA2* and *APC* somatic mutations affected RFS. These results indicate that sequential somatic mutations have a key role in drug resistance.

Several clinical and pathological factors, as shown in Table 1, were considered when analyzing the prognostic impact of risk groups stratified by sequential somatic mutations. The small sample size is a limitation of our study. However, our model showed a strong statistical significance for RFS (adjusted hazard ratio (HR) = 7.46, *p*-value = 0.000192). There are also some limitations in our methods. We did not consider the effect of copy number variants (CNVs), which will influence variant allele frequency (VAF). The number of SNVs and the distribution of VAFs in targeted deep sequencing may not be enough to represent our evolution model, and in terms of the case numbers, more data may be needed to make a strong conclusion.

## Conclusions

In conclusion, mutations in DNA damage response (DDR) genes represent promising biomarkers to assess the response of stage III CRC patients to oxaliplatin-based chemotherapy. The sequential order of and co-occurring DDR somatic mutations are associated with recurrence-free survival. We should consider the impact of tumor evolution when developing therapeutic strategies.

## Supplementary Information

**Additional file 1: Supplementary Fig. S1**. The association between mismatch repair and homologous recombination gene mutations. aHeatmap of CRC with MMR and homologous recombination genetic variants and clinical characteristics. bKaplan–Meier plot of MMR mutations in CRC patients, with no significant difference. **Supplementary Fig. S2**. Kaplan-Meier plot of decision subgroups T1, T2 and T3. Patients in T1 (*N* = 39) and T2 (*N* = 21) had longer recurrence-free survival than patients in T3 (*N* = 48). T1 vs T2 (HR = 1.05, 95% CI = 0.19–5.71, *P* = 1) T1 vs T3 (HR = 5.89, 95% CI = 1.99–17.4, *P* = 0.001) T2 vs T3 (HR = 5.75, 95% CI = 1.34–24.73, *P* = 0.02) (median recurrence-free survival, NA months [95% CI, NA-NA] vs 33.47 months [95% CI, 23.63-NA]. **Supplementary Fig. S3**. Time-dependent receiver operating characteristic (ROC) curves for 2, 5, 6, and 8 years before recurrence for the 2 groups assessed at follow-up visits. The area under the curve (AUC) of this model at 2, 4, 6, and 8 years in the training set was 0.73, 0.7, 0.797, and 0.88, respectively. **Supplementary Fig. S4**. PCA plot of subgroups G1 and G2 by sequential genetic mutations. There was a significant difference between G1 and G2 subgroups. Sequential germline (GL) genetic mutations followed by BRCA2, ATM and BRAF somatic mutations tend to be important features in G1; APC, TP53 and APC, NOTCH1 somatic mutations belong to G2. Red circles represent the G1 group and blue triangles the G2 group. **Supplementary Fig. S5** Heatmap ofCRCsequential genetic mutations andclinical characteristics occurringin at least 10 patients. **Supplementary Fig. S6**. Kaplan-Meier plot of ATM and TP53 with BRCA1 mutations of CRC patients in cBioPortal. aCRC patients with ATM mutations tend to have better survival than patients with ATM mutations. bThe occurrence of BRCA1 and TP53 somatic mutations led to a poorer prognosis than BRCA1 somatic mutations only in CRC patients. **Supplementary Table S1**. Patients characteristics in low and high risk groups. **Supplementary Table S2.**The association of MMR genes and HRD. **Supplementary Table S3.** ATM and BRCA2 mutations in subgroup C1 and C2. **Supplementary Table S4.** Patients characteristics in three groups.

## Data Availability

All data generated and analyzed during this study are presented in the Supplementary Information files of this manuscript. All sequencing datasets presented in this study can be found in online repositories. The names of the repositories/repositories and accession number(s) are as follows: NCBI BioProject (Accessions: PRJNA681983/ https://dataview.ncbi.nlm.nih.gov/object/PRJNA681983?reviewer=vocgf3viamqi24ehjp86kqlhvg).

## Abbreviations

BF: Bonferroni
CNV: Copy number variant
CRC: Colorectal cancer
DDR: DNA damage response
FA: Fanconi anemia
FDR: False discovery rate
HR: Hazard ratio
HR: Homologous recombination
IQR: Interquartile range
LASSO: Least absolute shrinkage and selection operator
MAF: Minor allele frequency
MMR: Mismatch repair
dMMR: Mismatch repair deficiency
MSI-H: Microsatellite instability-high
NCKUH: National Cheng Kung University Hospital
NER: Nucleotide excision repair
OCAs: Oncomine Comprehensive Assays
RPA: Recursive partitioning analysis
RFS: Recurrence-free survival
SNVs: Single-nucleotide variants
