# Supplementary material for: Sequential and co-occurring DNA damage response genetic mutations impact survival in stage III colorectal cancer patients receiving adjuvant oxaliplatin-based chemotherapy
Source: BMC Cancer. 2021 Mar 2;21:217. doi: 10.1186/s12885-021-07926-1 (PMC7923464; doi:10.1186/s12885-021-07926-1)
Supplement: Supplementary file 1 — Additional file 1: Supplementary Fig. S1. The association between mismatch repair and homologous recombination gene mutations. aHeatmap of CRC with MMR and homologous recombination genetic variants and clinical characteristics. bKaplan–Meier plot of MMR mutations in CRC patients, with no significant difference. Supplementary Fig. S2. Kaplan-Meier plot of decision subgroups T1, T2 and T3. Patients in T1 (N = 39) and T2 (N = 21) had longer recurrence-free survival than patients in T3 (N = 48). T1 vs T2 (HR = 1.05, 95% CI = 0.19–5.71, P = 1) T1 vs T3 (HR = 5.89, 95% CI = 1.99–17.4, P = 0.001) T2 vs T3 (HR = 5.75, 95% CI = 1.34–24.73, P = 0.02) (median recurrence-free survival, NA months [95% CI, NA-NA] vs 33.47 months [95% CI, 23.63-NA]. Supplementary Fig. S3. Time-dependent receiver operating characteristic (ROC) curves for 2, 5, 6, and 8 years before recurrence for the 2 groups assessed at follow-up visits. The area under the curve (AUC) of this model at 2, 4, 6, and 8 years in the training set was 0.73, 0.7, 0.797, and 0.88, respectively. Supplementary Fig. S4. PCA plot of subgroups G1 and G2 by sequential genetic mutations. There was a significant difference between G1 and G2 subgroups. Sequential germline (GL) genetic mutations followed by BRCA2, ATM and BRAF somatic mutations tend to be important features in G1; APC, TP53 and APC, NOTCH1 somatic mutations belong to G2. Red circles represent the G1 group and blue triangles the G2 group. Supplementary Fig. S5 Heatmap ofCRCsequential genetic mutations andclinical characteristics occurringin at least 10 patients. Supplementary Fig. S6. Kaplan-Meier plot of ATM and TP53 with BRCA1 mutations of CRC patients in cBioPortal. aCRC patients with ATM mutations tend to have better survival than patients with ATM mutations. bThe occurrence of BRCA1 and TP53 somatic mutations led to a poorer prognosis than BRCA1 somatic mutations only in CRC patients. Supplementary Table S1. Patients characteristics in low and high risk groups. [file 12885_2021_7926_MOESM1_ESM.pdf]

Sequential and co-occurring DNA damage response genetic mutations impact survival in stage III colorectal cancer patients receiving adjuvant oxaliplatin-based chemotherapy

Peng-Chan Lin[1,2,3], Yu-Min Yeh[1], Ren-Hao Chan[4], Bo-Wen Lin[4], Po-Chuan Chen[4], Chien-Chang Pan[2,3,7], Meng-Ru Shen[5,6,7*]

[1]Department of Oncology, National Cheng Kung University Hospital, College of Medicine, National Cheng Kung University, Taiwan

[2]Department of Genomic Medicine, National Cheng Kung University Hospital, College of Medicine, National Cheng Kung University, Taiwan

[3]Department of Computer Science and Information Engineering, College of Electrical Engineering and Computer Science, National Cheng Kung University

[4]Department of Surgery, National Cheng Kung University Hospital, College of Medicine, National Cheng Kung University, Taiwan

[5]Graduate Institute of Clinical Medicine, National Cheng Kung University Hospital, College of Medicine, National Cheng Kung University, Taiwan

[6]Department of Obstetrics and Gynecology, National Cheng Kung University Hospital, College of Medicine, National Cheng Kung University, Taiwan

[7]Department of Pharmacology, National Cheng Kung University Hospital, College of Medicine, National Cheng Kung University, Taiwan

[*]Correspondence to: Meng-Ru Shen, Department of Department of Obstetrics and Gynecology, National Cheng Kung University Hospital, College of Medicine, No.138, Sheng-Li Road, Tainan 704, Taiwan
Tel: +886-6-2353535 Ext 5505

Supplementary Fig. S1

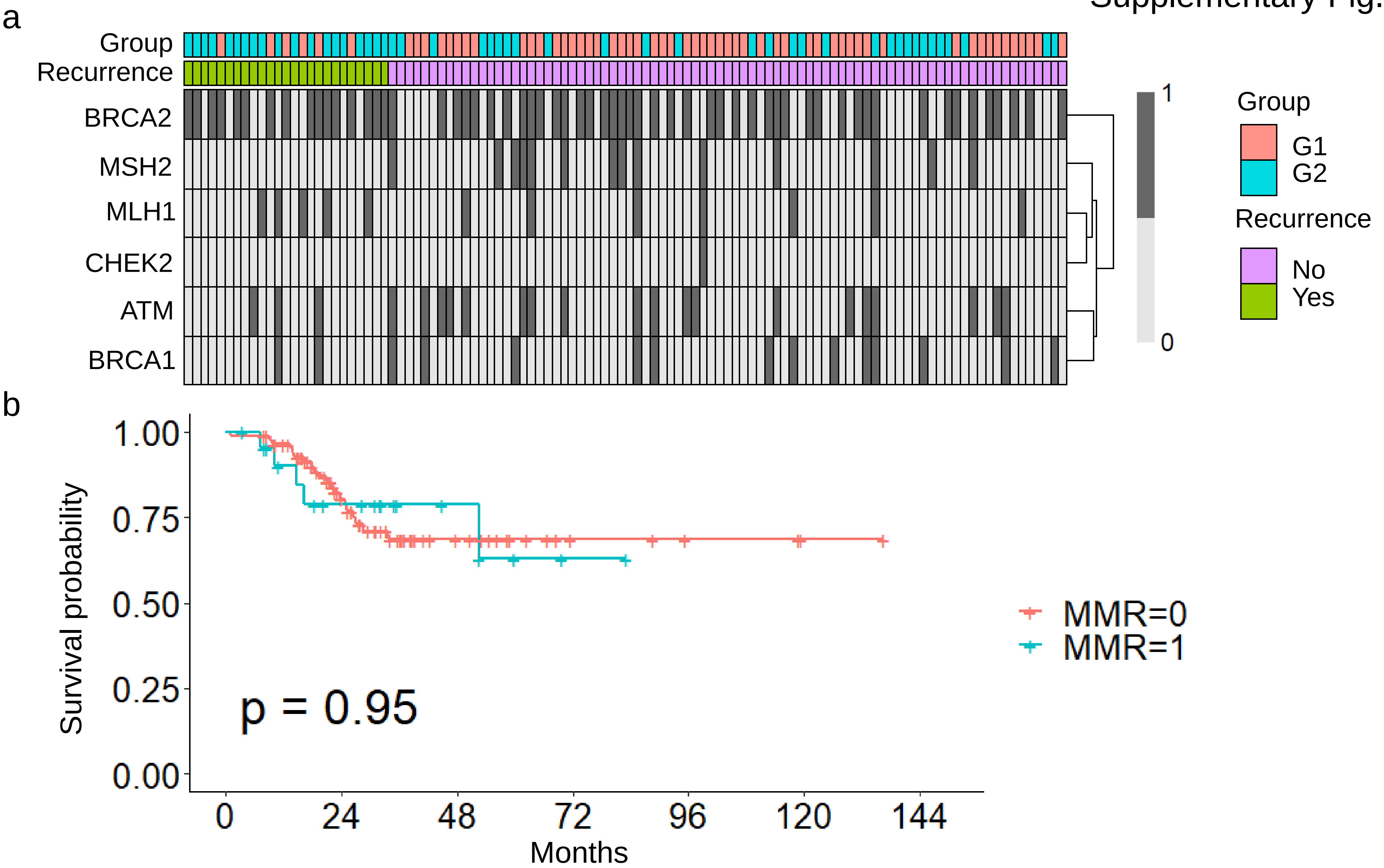

Supplementary Fig. S1 The association between mismatch repair and homologous recombination gene mutations. **a** Heatmap of CRC with MMR and homologous recombination genetic variants and clinical characteristics. **b** Kaplan–Meier plot of MMR mutations in CRC patients, with no significant difference.

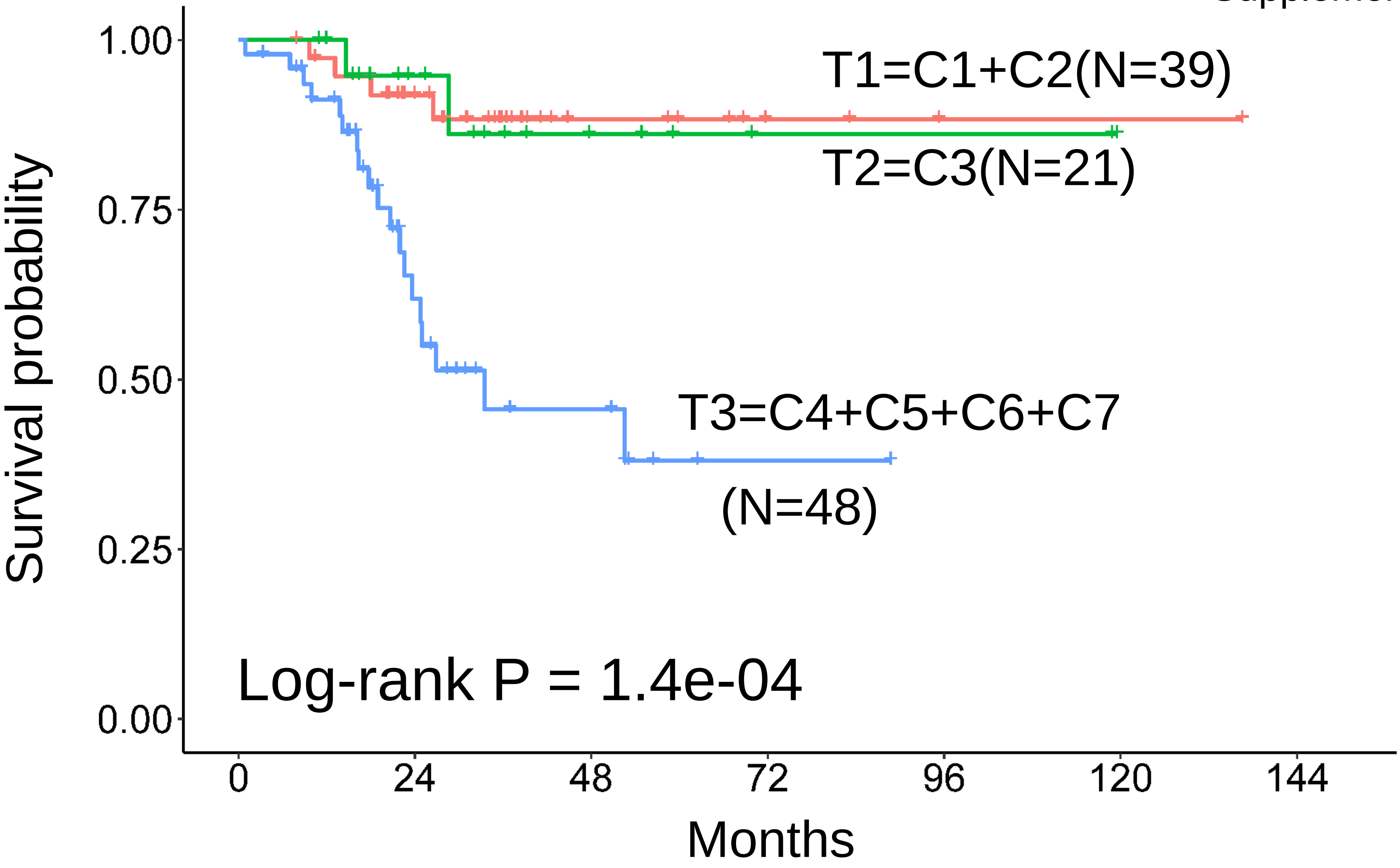

Supplementary Fig. S2 Kaplan-Meier plot of decision subgroups T1, T2 and T3. Patients in T1 (N = 39) and T2 (N=21) had longer recurrence-free survival than patients in T3 (N = 48). T1 vs T2 (HR = 1.05, 95% CI = 0.19–5.71, P = 1) T1 vs T3 (HR = 5.89, 95% CI = 1.99–17.4, P = 0.001) T2 vs T3 (HR = 5.75, 95% CI = 1.34–24.73, P = 0.02) (median recurrence-free survival, NA months [95% CI, NA-NA] vs 33.47 months [95% CI, 23.63-NA]

Supplementary Fig. S3

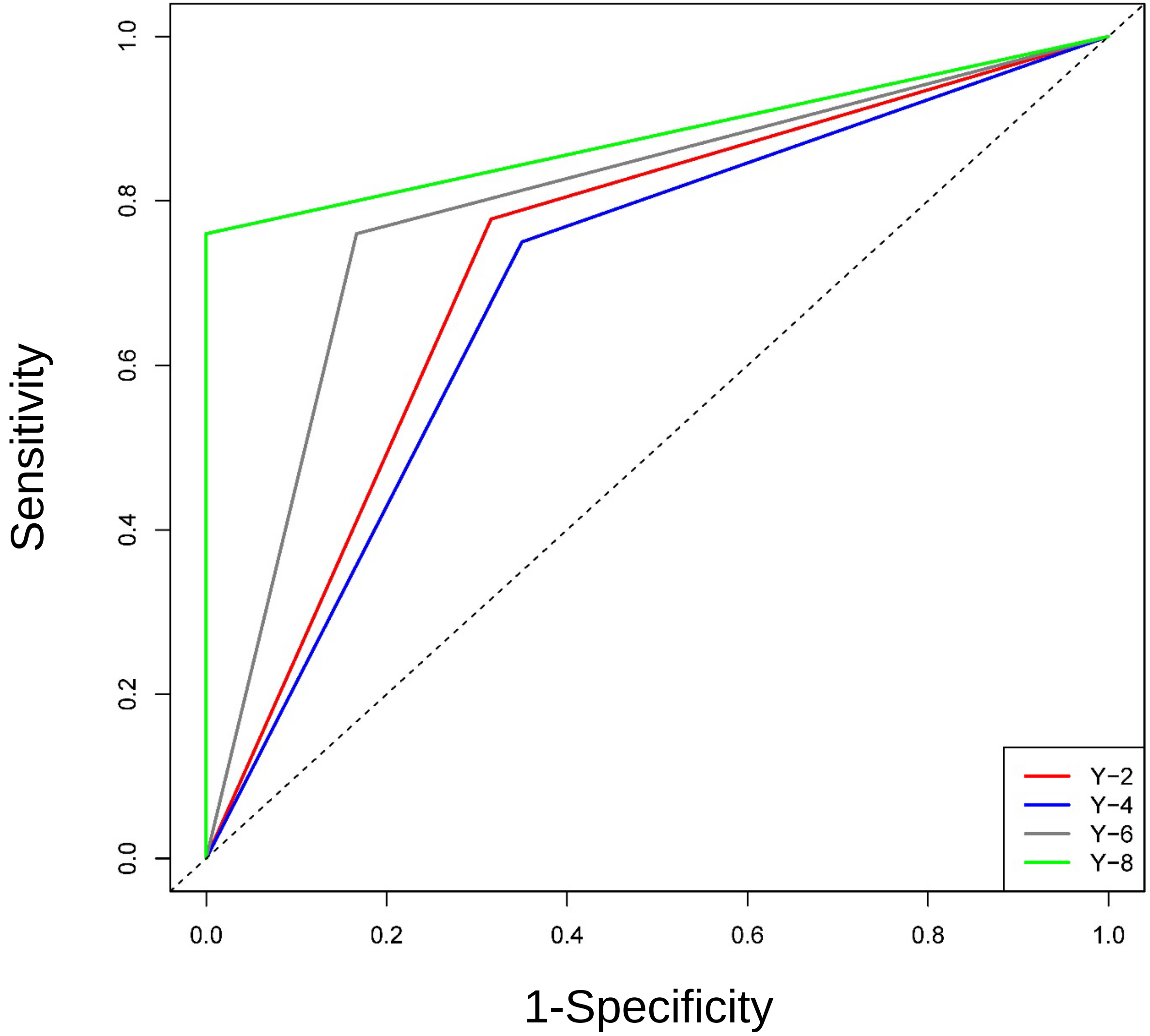

Supplementary Fig. S3 Time-dependent receiver operating characteristic (ROC) curves for 2, 5, 6, and 8 years before recurrence for the 2 groups assessed at follow-up visits. The area under the curve (AUC) of this model at 2, 4, 6, and 8 years in the training set was 0.73, 0.7, 0.797, and 0.88, respectively.

Supplementary Fig. S4

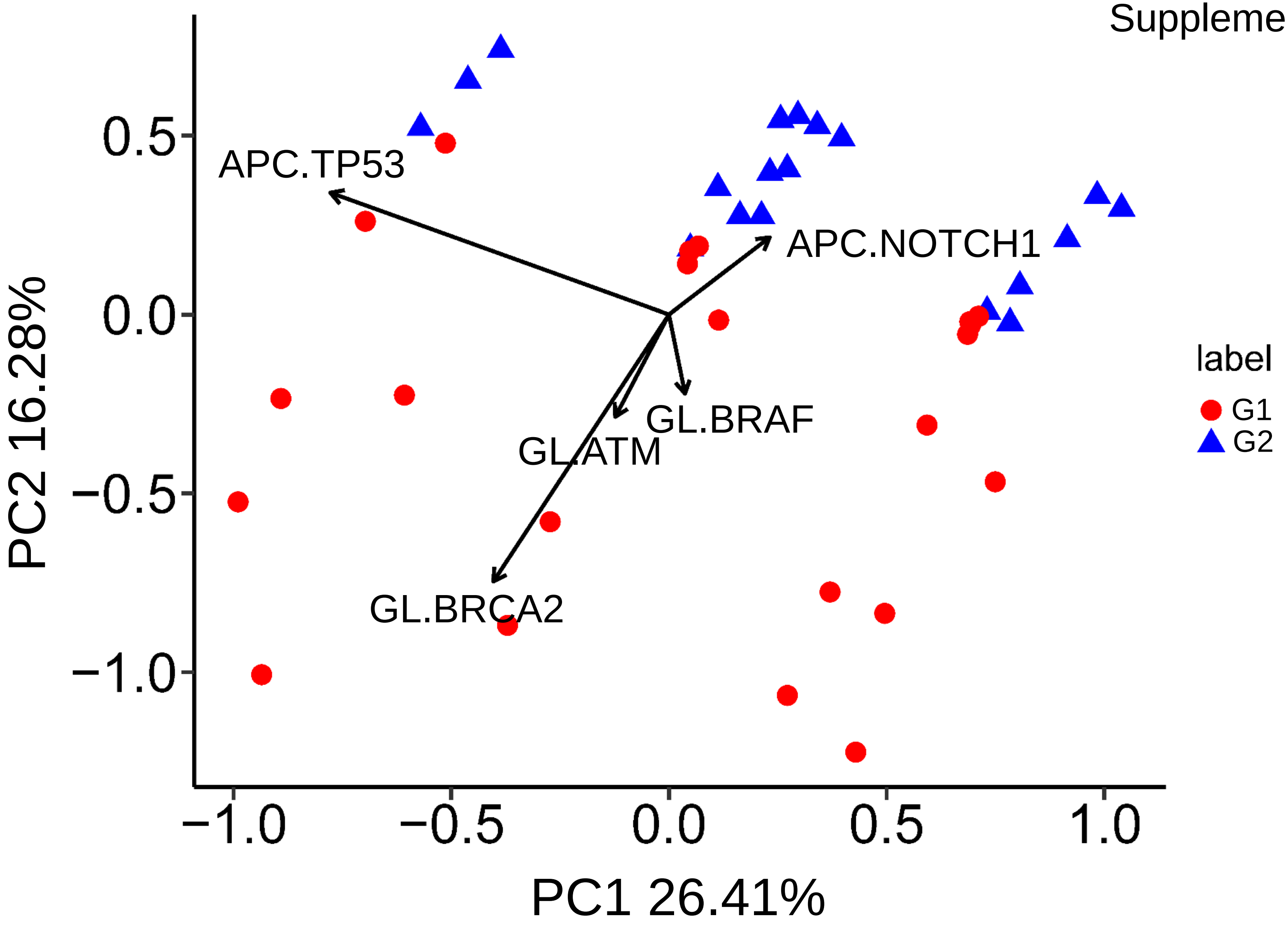

Supplementary Fig. S4 PCA plot of subgroups G1 and G2 by sequential genetic mutations. There was a significant difference between G1 and G2 subgroups. Sequential germline (GL) genetic mutations followed by BRCA2, ATM and BRAF somatic mutations tend to be important features in G1; APC, TP53 and APC, NOTCH1 somatic mutations belong to G2. Red circles represent the G1 group and blue triangles the G2 group.

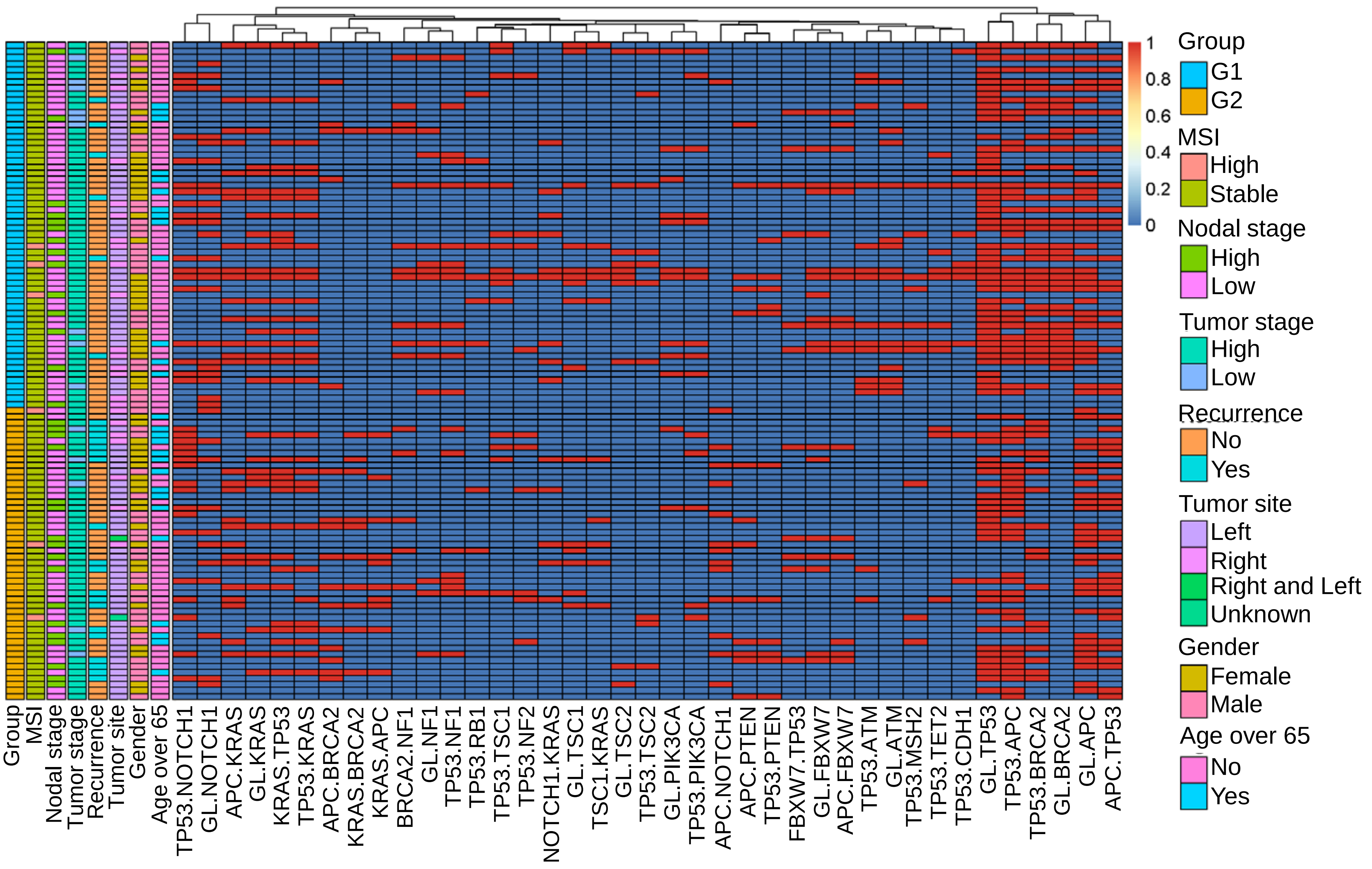

Supplementary Fig. S5 Heatmap of CRC sequential genetic mutations and clinical characteristics occurring in at least 10 patients.

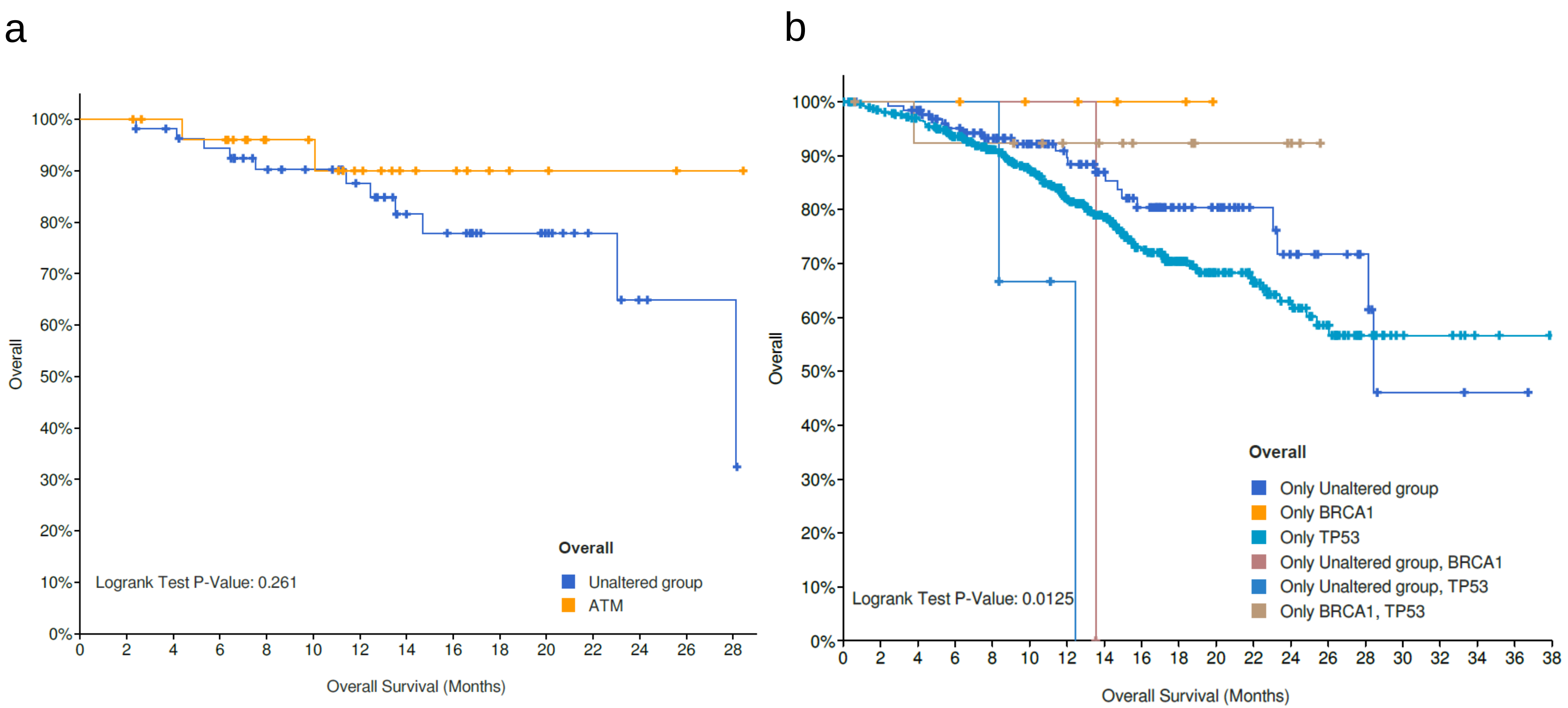

Supplementary Fig. S6. Kaplan-Meier plot of ATM and TP53 with BRCA1 mutations of CRC patients in cBioPortal. **a** CRC patients with ATM mutations tend to have better survival than patients with ATM mutations. **b** The occurrence of BRCA1 and TP53 somatic mutations led to a poorer prognosis than BRCA1 somatic mutations only in CRC patients.

Supplementary Table S1. Patients characteristics in low and high risk groups

Characteristic	All (N=108)	G1 (N=60)	G2 (N=48)	P value
Age				0.28
<65	80(74%)	47(78.3%)	33(68.8%)	
>=65	28(26%)	13(21.7%)	15(31.2%)	
Gender				0.85
Male	54(50%)	31(51.7%)	23(47.9%)	
Female	54(50%)	29(48.3%)	25(52.1%)	
Tumor site				0.16
Left	82(75.9%)	43(78.3%)	39(81.3%)	
Right	24(24.1%)	17(21.7%)	7(18.7%)	
Tumor invasion[a]				0.57
T1/T2	14(13%)	9(15%)	5(10.4%)	
T3/T4	94(87%)	51(85%)	43(89.6%)	
Lymph node[a]				0.2
N0/N1	77(71.3%)	46(76.7%)	31(64.6%)	
N2	31(28.7%)	14(23.3%)	17(35.4%)	
Mismatch Repair Status				0.99
Proficient	102(94.4%)	57(95%)	45(93.8%)	
Deficient	6(5.6%)	3(5%)	3(6.2%)	
MMR genetic variants				0.99
Mutation	22(20.4%)	12(20%)	10(20.9%)	
Wild Type	86(79.6%)	48(80%)	38(79.1%)	

Abbreviations:

DNA mismatch repair: MMR; mismatch repair status proficient: microsatellite instability high or MMR protein loss by Immunohistochemistry (IHC) statin.

a. The American Joint Committee on Cancer Stage

Supplementary Table S2.The association of MMR genes and HRD

A	B	Neither	A not B	B not A	Both	Odds Rati	p-Value	Tendency
MLH1	BRCA2	42	6	54	6	0.779612	0.762857	Mutual-exclusive
MLH1	ATM	79	7	17	5	3.272566	0.065713	Co-occurrence
MLH1	BRCA1	85	8	11	4	3.796234	0.061547	Co-occurrence
MSH2	BRCA2	44	4	50	10	2.1848	0.255471	Co-occurrence
MSH2	ATM	80	6	14	8	7.41203	0.00121	Co-occurrence
MSH2	BRCA1	83	10	11	4	2.977877	0.103713	Co-occurrence

Supplementary Table S3. ATM and BRCA2 mutations in subgroup C1 and C2

Chr	Start	End	Ref	Alt	Func.refGe	Gene.refG	ExonicFur	AAChange	Transcript	Exon
chr11	1.08E+08	1.08E+08	G	A	exonic	ATM	nonsynony	ATM:NM_	NM_0000	exon5
chr11	1.08E+08	1.08E+08	G	A	exonic	ATM	nonsynony	ATM:NM_	NM_0000	exon11
chr11	1.08E+08	1.08E+08	C	T	exonic	ATM	nonsynony	ATM:NM_	NM_0000	exon3
chr11	1.08E+08	1.08E+08	G	T	exonic	ATM	nonsynony	ATM:NM_	NM_0000	exon4
chr11	1.08E+08	1.08E+08	-	T	exonic	ATM	frameshift	ATM:NM_	NM_0000	exon25
chr11	1.08E+08	1.08E+08	G	A	exonic	ATM	stopgain	ATM:NM_	NM_0000	exon25
chr11	1.08E+08	1.08E+08	G	A	exonic	ATM	nonsynony	ATM:NM_	NM_0000	exon26
chr11	1.08E+08	1.08E+08	C	T	exonic	ATM	stopgain	ATM:NM_	NM_0000	exon7
chr11	1.08E+08	1.08E+08	C	T	exonic	ATM	nonsynony	ATM:NM_	NM_0000	exon28
chr11	1.08E+08	1.08E+08	C	T	exonic	ATM	stopgain	ATM:NM_	NM_0000	exon30
chr11	1.08E+08	1.08E+08	G	A	exonic	ATM	nonsynony	ATM:NM_	NM_0000	exon23
chr11	1.08E+08	1.08E+08	C	T	exonic	ATM	nonsynony	ATM:NM_	NM_0000	exon52
chr11	1.08E+08	1.08E+08	G	A	exonic	ATM	nonsynony	ATM:NM_	NM_0000	exon15
chr11	1.08E+08	1.08E+08	C	A	exonic	ATM	nonsynony	ATM:NM_	NM_0000	exon2
chr11	1.08E+08	1.08E+08	A	G	exonic	ATM	nonsynony	ATM:NM_	NM_0000	exon40
chr11	1.08E+08	1.08E+08	C	T	exonic	ATM	nonsynony	ATM:NM_	NM_0000	exon8
chr13	32915246	32915246	T	C	exonic	BRCA2	nonsynony	BRCA2:N	NM_0000	exon11
chr13	32906609	32906609	A	T	exonic	BRCA2	nonsynony	BRCA2:N	NM_0000	exon10
chr13	32914061	32914061	G	A	exonic	BRCA2	nonsynony	BRCA2:N	NM_0000	exon11
chr13	32905084	32905084	A	C	exonic	BRCA2	nonsynony	BRCA2:N	NM_0000	exon9
chr13	32907386	32907386	-	T	exonic	BRCA2	frameshift	BRCA2:N	NM_0000	exon10
chr13	32906873	32906873	G	A	exonic	BRCA2	nonsynony	BRCA2:N	NM_0000	exon10
chr13	32972340	32972340	A	T	exonic	BRCA2	nonsynony	BRCA2:N	NM_0000	exon27
chr13	32912073	32912073	G	T	exonic	BRCA2	nonsynony	BRCA2:N	NM_0000	exon11
chr13	32913785	32913785	A	C	exonic	BRCA2	nonsynony	BRCA2:N	NM_0000	exon11

AAChange
p.G138R
p.R568K
p.T39I
p.K93N
p.I1237fs
p.W1221X
p.M1321I
p.R248X
p.P1382L
p.Q1500X
p.R1106K
p.P2553L
p.S759N
p.H17N
p.Q1982R
p.R337C
p.S2252P
p.I332F
p.E1857K
p.D237A
p.I591fs
p.D420N
p.L3230F
p.G1194V
p.K1765Q

Supplementary Table S4. Patients characteristics in there groups

Characteristic		C1~2 (N=39)	C3 (N=21)	C4~7 (N=48)	P value
Age					0.35
	<65	32(82.1%)	15(71.4%)	33(68.8%)	
	>=65	7(17.9%)	6(28.6%)	15(31.2%)	
Gender					0.88
	Male	21(53.8%)	10(47.6%)	23(47.9%)	
	Female	18(46.2%)	11(52.4%)	25(52.1%)	
Tumor site					0.017
	Left	32(82.1%)	11(52.4%)	39(81.3%)	
	Right	7(17.9%)	10(48.6%)	7(18.7%)	
Tumor stage					0.58
	T1/T2	7(17.9%)	2(9.5%)	5(10.4%)	
	T3/T4	32(82.1%)	19(90.5%)	43(89.6%)	
Nodal stage					0.17
	N0/N1	32(82.1%)	14(66.7%)	31(64.6%)	
	N2	7(17.9%)	7(33.3%)	17(35.4%)	
Mismatch Repair Status					0.41
	Proficient	38(97.4%)	19(90.5%)	45(93.8%)	
	Deficient	1(2.6%)	2(9.5%)	3(6.2%)	
